# Conceptual construction and scale development of leadership taking charge behavior in the Chinese cultural context

**DOI:** 10.3389/fpsyg.2025.1578771

**Published:** 2025-08-06

**Authors:** Guanglin Bai, Shuhui Li, Yan Xu, Guijin Wang

**Affiliations:** School of Management, Jiangsu University, Zhenjiang, China

**Keywords:** taking charge, grounded theory, China, structural dimensions, scale development

## Abstract

This study aims to address the measurement issues related to leaders’ taking charge behavior within the context of Chinese culture and to fill the gap in the literature on taking charge behavior by developing and validating a scale for measuring this construct. In Study 1, a grounded coding method was used to construct a model of organizational identity structure and to preliminarily explore three dimensions of this behavior: initiative change, taking responsibility, and not fearing risks. In Study 2, exploratory factor analysis (*N* = 249) and confirmatory factor analysis (*N* = 244) were conducted to develop and validate a measurement scale with five items per dimension, confirming the best fit of a three-factor model. Additionally, to enhance the scale’s validity and practicality, three items with the highest factor loadings that best represent the core content of each dimension were selected to create a short version of the leadership taking charge behavior scale. Finally, Study 3 investigated the predictive utility of the short version of the leadership taking charge behavior scale by examining its relationships with two criterion variables: employees’ felt obligation for constructive change and their perception of organizational change significance. This study also further explored the impact mechanisms of leadership taking charge behavior.

## Introduction

Amid growing global economic and geopolitical uncertainties, rapid technological advancements, and the ongoing trend of de-globalization, organizations face heightened market competition and increasing environmental complexity and instability. In this turbulent context, change has become a critical factor for organizational survival and growth (Ma et al., 2023). Motivating organizational members to proactively initiate, engage in, and embrace change—thereby driving successful transformation—has garnered significant attention from both scholars and practitioners (Sun et al., 2022). Leaders, who are at the forefront of decision-making, play a key role in managing these changes by guiding employees and exemplifying desired behaviors. Their actions ultimately determine the success or failure of organizational transformation (Wang et al., 2023; He et al., 2023). To ensure that change achieves the desired outcomes, leaders must view it as a meaningful challenge or a positive opportunity, take responsibility for it, and act effectively. In this way, they can lead the organization to capitalize on opportunities amid challenges and achieve sustained development. In other words, leaders must embody taking charge behavior.

Taking charge refers to the voluntary, constructive efforts by individuals to initiate functional changes within an organization, aiming to improve the effectiveness of their roles, departments, or the organization as a whole (Morrison and Phelps, 1999). As a key strategy for navigating complex and volatile environments and enhancing organizational change effectiveness, this behavior has garnered considerable scholarly attention in recent years. A substantial body of research shows that in highly uncertain environments, taking charge behavior not only contributes to individual success and the formation of social networks but also enhances organizational adaptability and performance (Fuller and Marler, 2009). Additionally, it plays a significant role in promoting organizational adaptability and long-term survival (Xu et al., 2023). Current research on leadership taking charge behavior, both in domestic and international contexts, is rooted in the “taking charge” concept proposed by Morrison and Phelps (1999), which is grounded in Western cultural norms (Dai et al., 2021). These studies primarily focus on employees’ psychological mechanisms and behaviors when they respond proactively to change. However, they generally center on employees and utilize measurement scales developed by Western scholars, with limited research addressing leaders’ taking charge behavior (Dai et al., 2023). In organizations, leaders’ roles and statuses differ significantly from those of employees. Leaders are seen as initiators and promoters of change, wielding greater authority and influence in strategic formulation, decision-making, and resource allocation. As such, leaders bear broader and more extensive responsibilities than regular employees (Li et al., 2020). Consequently, leaders are in a relatively higher position regarding risk-taking and often face greater challenges during change initiatives. Therefore, leaders’ taking charge behavior may differ significantly from that of ordinary employees, emphasizing the importance of exploring leadership taking charge behavior in depth.

Moreover, current definitions and descriptions of taking charge behavior in the workplace largely stem from Western contexts. However, significant differences in values and ways of thinking exist between Eastern and Western cultures (Pan et al., 2014), which may lead to biased interpretations of the concept of taking charge behavior. Under the influence of traditional Chinese culture, the inherent characteristics of leadership behaviors and models exhibit unique qualities (Sun et al., 2020). This further underscores the need to consider cultural context when examining leadership taking charge behavior. Chinese society places a strong emphasis on “people-oriented” principles, with organizational management reflecting a human-centered approach (Liu et al., 2023), in stark contrast to the Western focus on individual rights and a rational, legalistic contract-based society. In this context, individuals’ destinies are often entrusted to authoritative figures. Therefore, leaders must internalize external responsibilities as moral imperatives, embracing the mission of “self-cultivation, managing the family, governing the country, and bringing peace to the world” (Cheng et al., 2021). This cultural expectation holds leaders accountable not only for promoting corporate development but also for ensuring employee well-being and societal stability, imposing greater pressure and challenges on them (Qin et al., 2023). If change efforts fail, leaders may suffer damage to their reputation, career setbacks, and even legal accountability. Additionally, within the relational and favor-based context of Chinese society, leaders often seek to establish broader social networks (Guo et al., 2017). However, this “relationship network” can create a ripple effect, where the implementation of change involves higher risks. Consequently, blindly applying the Western concept of taking charge behavior to Chinese organizational leaders, without accounting for cultural differences and contextual factors, may fail to accurately capture leadership taking charge behavior in China (Li et al., 2019).

In conclusion, while existing research on taking charge is extensive, there is relatively limited focus on “leadership taking charge behavior.” There is a lack of systematic exploration of its structural dimensions and the development of culturally appropriate measurement tools. Therefore, it is both theoretically and practically significant to explore the connotations and structural dimensions of leadership taking charge behavior within the Chinese cultural context and develop corresponding localized scales.

### Concept and characteristics of taking charge

The concept of “taking charge” was first introduced by Morrison and Phelps (1999), who defined it as the voluntary and proactive actions taken by employees to initiate functional changes within an organization aimed at improving work effectiveness. This definition has been widely accepted and expanded upon by scholars both domestically and internationally (Li et al., 2023). For instance, Choi (2007) further refined the concept, describing it as change-oriented organizational citizenship behavior. Taking charge, according to Choi, involves identifying and implementing changes in work methods, policies, and procedures—such as adopting improved processes, proposing new work methods, and correcting erroneous practices (Kim et al., 2023). This behavior may challenge the status quo, potentially leading to controversy and conflict (Kim et al., 2015).

Later scholars, focusing on motivation and role identity, have emphasized that taking charge embodies the essence of proactivity. It involves self-initiated, constructive, and anticipatory actions that aim to influence and modify the internal organizational environment (Parker and Collins, 2010; Parker and Wang, 2015). This reflects employees’ proactive efforts to implement positive changes in their work processes (Liu et al., 2022). Consequently, taking charge is classified as proactive work behavior. Recent research has also extended the concept to the work-family domain, where it is viewed as a resource-generating behavior. From the perspective of work flourishing, it is suggested that taking charge enables employees to create new resources through self-directed actions, contributing to a sense of flourishing (Xu et al., 2020).

In summary, although scholars have defined taking charge from various perspectives, all definitions converge on its core characteristics. Therefore, these definitions are not contradictory (Zhang and Li, 2019). A synthesis of the definitions reveals that the essential characteristics of taking charge are as follows:

*Spontaneity:* Taking charge is a self-initiated and self-determined behavior, rather than a formal directive from the organization (Kim and Liu, 2017).
*Change Orientation and Constructiveness:* It is focused on improvement and involves proposing or implementing constructive solutions to organizational challenges. The goal is to bring about functional changes in individual, team, or organizational performance, rather than simply maintaining the status quo (Morrison and Phelps, 1999).
*Challenging and Risky:* Taking charge involves challenging the existing organizational state, which may violate certain norms and carries a high level of uncertainty and risk (Parker and Collins, 2010). Moreover, due to varying organizational environments, taking charge may face resistance from peers or stakeholders, making it a potentially risky endeavor (Cangiano et al., 2021).

Moreover, “Taking Charge” is a proactive behavior closely related to transformational leadership. Transformational leadership involves leaders motivating and guiding employees to facilitate organizational change and achieve shared objectives. In contrast, taking charge behavior emphasizes leaders’ autonomous assumption of responsibility for driving change. Both reflect a positive intention and goal orientation toward transformation. Unlike transformational leadership, which focuses primarily on leader-subordinate interactions, taking charge highlights independent initiation and sustained effort in change processes. It demonstrates proactive responsibility and risk awareness in complex environments. Incorporating taking charge into the transformational leadership framework enhances understanding of leadership behaviors during organizational change, particularly in cultural contexts that emphasize leader initiative and accountability.

### Taking charge behavior of leaders in the Chinese cultural context

In traditional Chinese culture, the principle that “officials should dare to take the lead for the good of all under heaven” embodies a longstanding ethos of responsibility. This cultural norm is deeply embedded in societal expectations and forms a foundational basis for leaders’ taking charge behaviors. In China’s high power distance environment, ordinary employees have limited decision-making authority. Their roles in organizational change are typically restricted to micro-level responsibilities, such as adapting to new systems, learning procedures, executing tasks, and providing feedback. Furthermore, the prevailing business culture emphasizes obedience to superiors and respect for authority, reinforcing a cautious and deferential mindset among employees (Zhou and Liao, 2018). Consequently, employees often follow leaders’ directives passively during periods of change. In contrast, Chinese leaders possess greater authority and influence, along with elevated expectations from the organization, subordinates, and society (Qin et al., 2023). Influenced by Confucian values, leaders are morally expected to “lead by example and take initiative.” They are tasked with identifying the need for organizational change, mobilizing resources, managing stakeholder relationships, mitigating risks, and steering teams toward strategic transformation. This role reflects not only an ethical duty but also personal cultivation and a strong commitment to organizational purpose (Zhang et al., 2023).

Additionally, China’s guanxi-oriented social structure means that organizational change often extends beyond technical or procedural modifications. It can disrupt intricate relational networks (Guo et al., 2017). Leaders must maintain connections with a wide range of stakeholders—including government agencies, clients, and suppliers—while driving reform efforts. This interdependent structure, where “a single move affects the whole system,” amplifies both the complexity and uncertainty of implementing change. As a result, Chinese leaders’ taking charge behaviors are driven not only by moral obligation but also by deliberate, risk-conscious decision-making. They must balance potential benefits against various risks and demonstrate resilience and perseverance amid uncertainty. This is especially critical in high-stakes scenarios such as organizational restructuring, the adoption of key technologies, or the reallocation of interests. Leaders must consider impacts on performance, relational tensions, reputational risks, and personal career stability. Failure to execute change may lead to diminished authority, public scrutiny, or even legal consequences in extreme cases. Thus, taking charge entails not only moral courage and responsibility but also a pragmatic, context-sensitive approach to leadership under risk.

In contrast to Western contexts, which often emphasize institutional norms and individual agency, Chinese leaders’ taking charge behaviors arise from an internalized sense of moral duty and are enacted through proactive leadership and sustained effort in uncertain environments. Based on this understanding, the present study identifies three core features of taking charge behavior among Chinese leaders: initiative change, taking responsibility, and not fearing risks.

### Structure and measurement of leadership taking charge behavior

Although leadership taking charge behavior has not been directly explored in existing studies, both domestic and international scholars have conducted extensive research on its structure and measurement. Morrison and Phelps (1999) were the first to define the unidimensional structure of taking charge and developed a 10-item scale to measure behaviors aimed at improving workplace procedures, methods, and policies. Since then, various scholars have created shortened and revised versions of this scale to meet the specific needs of their research. For instance, Parker and Collins (2010) developed a three-item unidimensional scale to differentiate various proactive behaviors, including taking charge. Harrison et al. (2011) refined and integrated three items from the original scale to better suit new employees, narrowing the scope of two remaining items, which resulted in a seven-item shortened scale. In a similar vein, several Chinese scholars have selected high-factor loading items from the taking charge scale as measurement standards. For example, Hao and Long (2020) and Dai et al. (2021) used a revised version of the scale, incorporating high-factor loading items such as “attempting to improve group or departmental work procedures,” “attempting to correct a faulty step or work pattern,” and “proposing solutions to urgent issues within the company.”

In summary, current research on taking charge reveals several key points: First, with regard to its dimensional structure, taking charge is generally considered unidimensional, although it likely encompasses multiple dimensions (Li et al., 2019). Limiting taking charge to a single dimension may hinder a full understanding of the behavior and its empirical validity. Second, while existing scales primarily emphasize the “change” aspect of taking charge, they often overlook other critical characteristics such as autonomy, challenge, and risk, which are integral to the behavior (Deng and Liu, 2021). Additionally, most studies have relied on the 10-item scale developed by Morrison and Phelps, or its shortened versions, which are based on Western contexts and focus on employee behavior. These studies tend to neglect leadership characteristics, and the applicability of the scale to leadership behavior in the Chinese cultural context remains unverified (Dai et al., 2019). In conclusion, the dimensional structure and measurement of leadership taking charge behavior require further investigation. It is crucial to explore the dimensionality of leadership taking charge behavior within the Chinese cultural context and to develop an appropriate scale for this purpose.

## Study 1: exploration of the dimensional structure of leadership taking charge behavior

### Research methodology

The grounded theory research method, introduced by American scholar Glaser in 1967 (Glaser et al., 1968), is a bottom-up approach for developing substantive theory. This method involves systematically collecting data to identify core concepts that capture the essence of phenomena and constructing theories based on the relationships among these concepts. By minimizing researchers’ subjective biases and preconceived notions, it is particularly well-suited for exploratory research in emerging fields. As a result, it is commonly used by scholars in conceptual development. In this study, we utilized NVivo software to systematically analyze extensive primary data, aiming to explore the conceptual structure of leadership taking charge behavior from the ground up.

### Data collection

Given the rigorous data requirements of grounded theory, this study combined both primary and secondary data collection methods, including open-ended questionnaires, in-depth interviews, and literature reviews.

#### Open-ended questionnaire survey

The primary objective of the open-ended questionnaire survey was to gather respondents’ insights into leadership taking charge behavior, which would aid in constructing this concept. The questionnaire focused on the definition of change-oriented leadership and included specific questions such as: “How do you define taking charge?” “What specific behaviors do you think a leader with taking charge would exhibit in their work and life?” and “Based on your experiences, describe your understanding of a leader’s taking charge behavior (at least five points).” The survey was administered both online and offline. The online portion was conducted via SoJump and distributed through social media channels like WeChat and QQ, targeting employed individuals. The offline portion focused on MBA and MPA students. A total of 115 questionnaires were distributed, with 101 returned, yielding 86 valid responses. The respondents included 49 males and 37 females.

#### In-depth interviews

Data collection through semi-structured interviews provided deeper insights into respondents’ perceptions of change-oriented leadership. The interviews were conducted both face-to-face and by telephone. The interview guide was designed to be clear and logically structured, ensuring that questions were comprehensible and coherent, which allowed respondents to express their views and experiences smoothly. Specific questions included: “How do you understand leadership taking charge behavior?” “Have you observed any leaders exhibiting this behavior in your workplace? If so, what were the specific manifestations?” “Do you admire or idolize any particular leader? Does this leader demonstrate taking charge behavior?” and “Can you provide detailed examples of taking charge behavior as you understand it?” Thirteen enterprise managers were interviewed, including seven males and six females, with an average of 8.4 years of work experience. With the interviewees’ consent, all interviews were recorded and transcribed, resulting in approximately 30,000 words of transcribed text.

#### Literature review

Using publicly available secondary data for academic research is widely recognized as a valuable practice. It can be integrated into the coding process as original qualitative material, complementing primary data and enhancing the accuracy of results. In this study, we collected literature, biographies, and relevant materials on leadership taking charge behavior from CNKI, published books, and online media sources. These materials were summarized and analyzed, yielding over 15,000 words of textual data. The qualitative data imported into NVivo for analysis included 86 open-ended questionnaires, 13 interview transcripts, and relevant secondary data, totaling approximately 51,200 words. The data were randomly divided into two groups. Two-thirds of the open-ended questionnaires, interview transcripts, and literature materials—comprising 57 open-ended questionnaires, nine interview transcripts, and approximately 10,000 words of summarized text—were selected for model construction. The remaining data were reserved for testing the theoretical saturation of the model.

The study first involved collecting literature materials and interview texts. Next, the subjects were randomly grouped into experimental and control groups, with two-thirds of the total sample selected for detailed analysis. The open-ended questionnaires captured participants’ genuine thoughts, while the interview texts provided deeper insights into their perspectives. All data were integrated to form the research conclusions.

### Exploration of the structure of leadership taking charge behavior

#### Open coding

Open coding involves the systematic conceptualization and categorization of raw data. Initially, interview texts are coded at the word, sentence, and paragraph levels using a browsing-based coding method to generate free nodes. To minimize subjective bias, two researchers independently perform the coding in the early stages. Afterward, their coding results are compared and discussed. A secondary verification process is then conducted using three criteria: typicality, accuracy, and relevance. The research team reviews the original texts and audio recordings, removing codes that deviate from core topics, have low frequency, or are semantically ambiguous, while consolidating redundant codes with overlapping meanings. Following this process and in-depth discussion, 23 open coding nodes were identified (see Table 1).

**Table 1:** Example of partial open coding results for Subject A.

Source material	Free nodes
Even when challenged by uncertainty, resulting in a strategic plan that fails to achieve the desired goals, A remains steadfast and shares the results with the organization;	Suffer the consequences
In the face of some opposition, A is able to hold on to his personal beliefs and push forward when change is blocked, ensuring that meaningful change is achieved through clear thinking about change;	Taking the heat
In the course of work, A frequently encountered situations where his personal production tools were borrowed, which inadvertently increased the number of machine parameterization problems caused by the exchange. Therefore, during the process of coding procedures, some of the procedures would be standardized, and at the same time, an exhaustive annotation strategy was adopted to improve the overall productivity. At the same time this will also influence the company’s procurement procedures to a high level of automated coding, not only to enhance the efficiency of work, but also greatly enhance the stability and controllability of the overall production process;	Proactive problem solving
When encountering moments of conflict or even crisis, A is brave enough to look at it correctly, move forward and swim against the current;	Not being afraid of difficulties and obstacles
A dares to make the decision to change, and is steadfast and fearless in the process;	Courage to change
A dares to challenge the existing traditional work habits and ways of thinking, the courage to break the routine;	Breaking the status quo
When faced with change, A is brave enough to bear the pressure, stand up for himself, move forward with determination, and persevere to the end;	Resisting Pressure
A possesses the spirit of ownership, actively contributes, takes the initiative to adapt to the environment, and has the courage to explore and innovate;	Leading by example
When encountering the wrong opinion of the superior audit, A does not just obey, rationally accuses the bias, according to the facts to fight;	Dare to face resistance

#### Axial coding

This study classifies and integrates 23 free nodes based on the results of open coding. Through iterative analysis, it identifies the correlations, hierarchical relationships, and identity features between concepts, using semantic association, process logic, and result orientation. NVivo software’s clustering analysis function is employed to generate a dendrogram based on semantic similarity, which reveals the potential connections between nodes. A systematic comparison is then performed to refine the main and subcategories. For example, nodes such as “actively change” and “propose new work methods” are grouped under the main category “active transformation.” In conclusion, the study establishes three category frameworks (Table 2), with each category derived from semantic integration and logical association, ensuring strong alignment with the research theme.

**Table 2:** Axial coding results.

Main category	Sub-category	Connotation of category
Initiative change	Take Initiative to change	Initiating and implementing change voluntarily, rather than passively accepting instructions
	Propose new working methods	Innovating or introducing new work processes, technologies, or methods to improve work efficiency
	Improve organizational content	Optimizing internal elements of the organization, such as structure, culture, policies or procedures, in order to improve the operational efficiency of the organization
	Innovation management system	Developing or adopting new management strategies and systems, introducing advanced technological tools and optimizing management processes
	Mode of innovation	Relying on innovation to realize the development of business models, operational strategies or thinking frameworks
	Lead by example	Inspire and guide others to participate actively in change by setting an example through their own behavior and attitudes.
	Take the Initiative to solve problems	Proactively identifying and responding to problems and challenges that arise during the process of organizational change, and ensuring the smooth progress of the change by adopting timely and effective solutions.
	Curb the old system	Eliminate or update outdated work systems, technologies, and processes to improve organizational adaptability.
	Error correction	Identify and correct errors and deviations in the process of organizational change in a timely manner to ensure that the change can maintain the correct direction of the established goals
	Breaking the status quo	Willing to challenge and change existing ways of thinking and work habits, and inspire others to go beyond the status quo to achieve deep transformation and development.
	Be perceptive	Sensitive to changes in the internal and external environments of the organization, quickly identifies needs and potential opportunities for change, and provides strategic recommendations to the organization.
Taking responsibility	Assume and be responsible for	Willingness to take responsibility for the change process and implement the necessary actions to see the change through to completion
	Suffer the consequences	Willingness to accept the positive and negative consequences of change, including personal gains and losses, organizational restructuring, and potential risks.
	Bear the pain	Understand and tolerate short-term discomfort, disruption or challenges that may be encountered during the change process.
	Bear the blame	Hold fast to the belief in change in the face of criticism, skepticism, or lack of understanding.
	Actively Responding to Problems	Consciously seeks appropriate responses and solutions to problems and challenges that arise during the change process.
	Be bold	Courage to make decisions about change and take responsibility for the consequences, without avoiding responsibility.
Not fearing risks	Take risks	Courage to face the uncertainty of change and dare to take actions that may be risky but beneficial to the organization.
	Stand up to resistance	Courage to deal with resistance and opposition to change within the organization.
	The compressive	Remain calm and focused under the pressure and challenges of change, without external interference, and demonstrate strong mental toughness and resilience.
	Not afraid of difficulties and dangers	Remaining steadfast and optimistic in the face of difficulties and obstacles in the change process
	Brave change	Determined to drive and implement change even in the face of uncertainties and challenges.
	Overcome difficulties	Takes effective measures to continually solve problems and meet challenges

#### Selective coding

Selective coding builds upon axial coding by further integrating and refining core categories. Its purpose is to clarify the internal relationships between the main and secondary categories and to construct the core framework of the theoretical model. Through repeated analysis and comparison of the raw data, the core category of “leadership change responsibility behavior” was identified. Three key dimensions emerged: proactive change, assuming responsibility, and risk-taking. To ensure scientific rigor and reliability, the research team carefully analyzed and reclassified the 23 subcategories generated during the axial coding phase. They also reviewed extensive literature on leadership change and responsibility behavior to support the development of theoretical insights. When the coding results stabilized and no new concepts emerged, data saturation was reached, signaling the completion of the coding process. Ultimately, three core categories closely related to leadership change responsibility behavior were identified.

### Ensuring reliability and validity of the coding

In this study, two independent coders performed the initial coding. Upon completion, we conducted consistency checks on their results. Following Tang et al. (2015), a consistency score between 0.61 and 0.80 indicates a high degree of agreement between two or more researchers. Using their method, we obtained a reliability score of 0.75, which exceeds the 0.6 threshold. To assess theoretical saturation, we applied the same open coding process to the second set of data as was used for the first. The results showed that all data could be classified into the existing three main categories, with no new subcategories emerging. Therefore, we concluded that the study’s results are reliable and that the model of leadership taking charge behavior has reached theoretical saturation. In summary, the coding results demonstrate both strong reliability and validity.

## Study 2: development and validation of the leadership taking charge behavior measurement scale

### Development of the initial scale

During the initial item compilation, we extracted statements from the raw data that aligned with the core content of the three identified dimensions. Additionally, we consulted items from relevant existing scales, systematically processing and organizing these to develop the preliminary items for the leadership taking charge behavior measurement scale. We ensured that each dimension included more than five measurement items, resulting in a total of 26 preliminary items.

To ensure the concept of leadership taking charge behavior accurately reflected organizational practice, we validated it using data from 10 in-depth interviews. After constructing the preliminary scale, the research team meticulously reviewed and discussed each item, consolidating redundant items and removing those that did not align with the core concept or were poorly worded. This process led to the retention of 18 items, which were distributed across the dimensions as follows: 8 items for proactive change, 5 for taking responsibility, and 5 for risk-taking.

Next, we formatted the items into a questionnaire using a 5-point Likert scale. External experts and research team members participated in a pilot test and evaluation of the questionnaire. Items that were controversial or ambiguous were specifically highlighted, and feedback was solicited. This feedback confirmed that all items were clearly understood and did not raise any objections. Following this, the research team conducted further discussions, incorporating suggestions from experts, peers, and other relevant parties to refine some of the phrasing. The final result was an initial measurement questionnaire for leadership taking charge behavior, consisting of 3 dimensions and 18 items.

### Formal survey

#### Participants and procedure

This study focused on employed individuals and adopted a mixed-methods approach to data collection, considering the characteristics of the research subjects. Online data were primarily collected through SoJump, utilizing a snowball sampling method to reach corporate employees. Offline data were gathered through paper questionnaires administered to MBA students. A total of 680 questionnaires were distributed, of which 605 were returned. After excluding 112 invalid responses, 493 valid questionnaires were retained, resulting in a valid response rate of 72.5%. The sample distribution is as follows: 51.32% female and 48.68% male participants. The age distribution of participants was: 28.60% were 25 years old or younger, 51.93% were between 26 and 35 years old, 16.02% were between 36 and 45 years old, and 3.45% were 46 years old or older. In terms of education, 8.52% had a degree below a bachelor’s, 59.43% held a bachelor’s degree, 29.61% had a master’s degree, and 2.43% had a doctorate or higher. Regarding work experience, 28.19% had less than 1 year, 33.47% had between 1 and 3 years, 14.20% had between 4 and 6 years, and 24.14% had more than 6 years of experience.

For the exploratory and confirmatory factor analyses, the 493 valid questionnaires were randomly divided into two independent datasets: Group A, consisting of 249 questionnaires, was used for exploratory factor analysis (EFA), while Group B, comprising 244 questionnaires, was used for confirmatory factor analysis (CFA).

#### Exploratory factor analysis

We conducted the Kaiser-Meyer-Olkin (KMO) test and Bartlett’s test of sphericity on the Group A sample data using SPSS 20.0. The results revealed a KMO value of 0.958 (*p* < 0.001) and a chi-square value of 4278.855 (df = 105, *p* = 0.000) for Bartlett’s test, indicating that the data were suitable for exploratory factor analysis. Next, we performed principal component analysis with varimax rotation. Applying the eigenvalue criterion of greater than 1, supplemented by scree plot analysis, we extracted three core factors.

To ensure a valid and interpretable factor structure, we followed these criteria: (1) eliminating items with factor loadings below 0.50, (2) maintaining consistency in item content within each factor, and (3) ensuring each factor contained at least three items. As a result, 15 items were retained, and the scale explained a cumulative variance of 82.47% (see Table 3). These findings demonstrate a clear and robust factor structure for the leadership taking charge behavior scale, supporting its validity and reliability for future research.

**Table 3:** Results of the exploratory factor analysis (*N* = 249).

Items	Factor structure		
	F1	F2	F3
Q3: My leader is the first to adopt new techniques or methods to improve work efficiency	0.784		
Q4: My leader is willing to break your work habits or ways of thinking	0.760		
Q5: My leader is willing to go the extra mile to effect change	0.725		
Q6: My leader Often corrects faulty procedures or practices at work	0.794		
Q7: My leader Constantly improves the organization’s work procedures to improve the effectiveness of work	0.797		
Q9: My leader is willing to bear the pain of change		0.688	
Q10: My leader dares to accept the consequences of failure to change		0.699	
Q11: My leader actively addresses issues that arise during the change process.		0.740	
Q12: When change is in danger, My leader stands up		0.719	
Q13: My leader confronts problems in reform and not avoid them		0.635	
Q14: My leader is willing to take risks for change.			0.783
Q15: My leader is not afraid of failure in change.			0.831
Q16: My leader dares to face colleagues’ or superiors’ doubts or opposition to change.			0.662
Q17: My leader can withstand pressure and persist in carrying out change to the end.			0.652
Q18: My leader, aware of the risks of change, still bravely moves forward.			0.771

Factor name	Initiative change	Taking responsibility	Not fearing risks
Eigenvalue	4.431	4.244	3.696
Contribution rate (%)	29.539	28.293	24.640
Cumulative contribution rate (%)	29.539	57.832	82.472

#### Confirmatory factor analysis

We conducted a confirmatory factor analysis (CFA) using data from Group B (*N* = 244) and employed MPLUS software to test the structural model of leadership taking charge behavior. A model comparison strategy was applied, involving a null model (assuming that the 15 items of leadership taking charge behavior do not share common factors) and four alternative models, each constructed from different combinations of items representing the three dimensions.

The comparison of fit indices showed that the three-factor model fit the data better than the one-factor and two-factor models (Table 4). Specifically, the fit indices for the three-factor model were: χ^2^ = 204.758, df = 84, χ^2^/df = 2.438, RMSEA = 0.077, SRMR = 0.019, CFI = 0.976, and TLI = 0.970. All indices met the recommended standards, confirming the validity of the three-factor model of leadership taking charge behavior and demonstrating its structural stability and rationality.

**Table 4:** Fit statistics of competitive models (*N* = 244).

Model	χ^2^	df	χ^2^/df	RMSEA	CFI	TLI	SRMR
Null Model	1132.103	105	10.782	0.400	0.000	0.000	0.490
Single-factor model (Tc + Tr + Nr)	631.225	90	7.014	0.157	0.891	0.873	0.037
Two-factor model (Tc + Nr; Tr)	522.352	89	5.869	0.141	0.913	0.897	0.034
Two-factor model (Tr + Nr; Tc)	515.185	89	5.789	0.140	0.914	0.899	0.034
Two-factor model (Tc + Tr; Nr)	392.770	89	4.413	0.118	0.939	0.928	0.023
Three-factor model (Tc; Tr; Nr)	204.758	84	2.438	0.077	0.976	0.970	0.019

T_c_ represents “Initiative change,” T_r_ represents “Taking responsibility,” and N_r_ represents “Not fearing risks.”

Next, we assessed the reliability of the scale, focusing on Cronbach’s *α* coefficient and the corrected item-total correlation (CITC) (Table 5). The results showed that the reliability coefficients for the dimensions of initiative change, taking responsibility, and not fearing risks were 0.943, 0.957, and 0.955, respectively. Removing any item would reduce the reliability of the corresponding factor. The overall reliability of the scale was 0.977, indicating excellent internal consistency both within each of the three factors and across the entire scale. Additionally, the CITC test evaluates the relationship between individual items and the overall scale. Items with a CITC value below 0.5 indicate low significance to the overall scale. All items in this study’s leadership taking charge behavior scale had CITC values exceeding 0.8, demonstrating the scale’s high reliability.

**Table 5:** Correlation coefficients and AVE values of factors (*N* = 244).

Factor	Initiative change	Taking responsibility	Not fearing risks
Initiative change	(0.877)		
Taking responsibility	0.836^**^	(0.906)	
Not fearing risks	0.814^**^	0.894^**^	(0.899)
Cronbach’s α	0.943	0.957	0.955
CR	0.943	0.958	0.955

^**^Indicates *p* < 0.01; the diagonal values in parentheses are the arithmetic square roots of the AVE for each factor.

Validity testing focused on content validity, convergent validity, and discriminant validity. Initially, expert judgment was used to evaluate content validity. The scale was developed based on existing literature on taking charge behavior and refined through in-depth interviews with academic experts and employed personnel. A pretest of the measurement items was conducted, and feedback was collected to further refine the scale by merging redundant items and removing unsuitable ones, ensuring rigorous and reliable content.

Convergent validity was assessed using composite reliability (CR) and average variance extracted (AVE). The results showed that all item factor loadings exceeded 0.6 and were statistically significant. The CR values for the three factors were 0.943, 0.958, and 0.955, all exceeding the threshold of 0.8. Additionally, the AVE values for these factors were greater than 0.6, confirming strong convergent validity. For discriminant validity, the square roots of the AVE values for each factor were compared with the correlation coefficients between factors. Each factor’s AVE square root was higher than its correlations with other factors, demonstrating robust discriminant validity.

To enhance the validity and practicality of the scale, this study selected the three items with the highest factor loadings from each dimension, ensuring that they best represented the core content of each factor. This resulted in the creation of a shortened version of the Leadership Taking Charge Behavior Scale. Confirmatory factor analysis was conducted to validate the factor structure of the brief version. As shown in Table 6, the fit indices for the factor model of the brief version (Model 2) were superior to those of the 15-item, 3-factor model (Model 1). Consequently, the Leadership Taking Charge Behavior Scale developed in this study consists of three dimensions—initiative change, taking responsibility, and risk-taking—comprising 3 factors and 9 items.

**Table 6:** Model fit indices (*N* = 244).

Model	χ^2^	df	χ^2^/df	RMSEA	CFI	TLI	SRMR
Model 1	278.807	87	3.205	0.095	0.961	0.953	0.0208
Model 2	39.562	24	2.420	0.067	0.965	0.956	0.0155

## Study 3: a study of the predictive utility of the leadership taking charge behavior scale

### Research hypotheses

The primary criterion for evaluating the validity of a measurement tool is determining whether the variables it measures significantly influence outcomes as predicted by theoretical expectations. To further establish the scale’s validity, it is crucial to construct a relevant theoretical model. Social learning theory suggests that human behavior is primarily learned through the observation and imitation of trusted role models (Bandura, 1977). According to this theory, leaders who exhibit taking charge behaviors can inspire employees’ enthusiasm for change by serving as role models. This modeling motivates employees to view innovation and change as personal responsibilities and helps them recognize the importance of change for both individuals and the organization. Consequently, employees develop strong internal beliefs about change and responsibility, which significantly impact their sense of accountability for constructive change and shape their perception of its importance within the organization.

Evidence shows a close relationship between taking charge and constructive change responsibility (Gao, 2023), and leaders who demonstrate such behavior can improve employees’ perceptions of organizational change (Dai et al., 2023). Based on these findings, this study uses employees’ sense of obligation for constructive change and their perception of the significance of organizational change as validity variables. This approach aims to further assess the predictive validity of the Leadership Taking Charge Behavior Scale and explore the mechanisms through which leadership taking charge behavior influences these variables.

#### Leadership taking charge behavior and employees’ felt obligation for constructive change

The close working relationship between leaders and employees positions leaders as key role models and objects of observation in daily interactions. Research shows that positive leadership styles, particularly those focused on change, significantly enhance employees’ motivation through social learning and imitation. This increased motivation, in turn, improves work performance and cognitive abilities (Liu et al., 2024). Positive leadership behaviors not only foster an inclusive, sustainable, and dynamic work environment but also serve as an interactive learning model. These behaviors shape and reinforce employees’ perceptions and attitudes, cultivating a strong sense of identity and responsibility within the organization. This dynamic interaction enhances employee engagement, commitment, and overall alignment with organizational goals (Waqas et al., 2021).

Felt obligation for constructive change refers to the belief that individuals are responsible for facilitating beneficial change (Babalola et al., 2021). This concept implies that employees must see themselves as accountable for implementing positive changes within the organization, motivating them to take proactive actions to safeguard organizational interests (Sarkar et al., 2022). According to social learning theory, leaders play a critical role in shaping the organizational environment and influencing social interactions, significantly impacting employees’ sense of responsibility and their beliefs about change (Zhang and Liu, 2020).

In the Chinese cultural context, leadership taking charge behavior focuses on responsibility and encompasses three dimensions: initiating change, assuming responsibility, and embracing risk. This behavior consistently emphasizes self-awareness and a sense of duty throughout the change process. Leaders who excel at managing change perform exceptionally well during challenging times. Their success not only earns employee recognition but also inspires emulation, fostering similar attitudes and behaviors among employees (Wang et al., 2024). Through visionary incentives and the demonstration effect, leaders motivate employees to adopt responsibility for change as their own. Employees begin to view initiating or participating in organizational change as an obligation, driving them to actively contribute to organizational development and improvements. Ultimately, this nurtures a strong belief in the importance of constructive change and reinforces a deep sense of responsibility for it.

Therefore, we hypothesize that leadership taking charge behavior positively has a positive effect on employees’ felt obligation for constructive change (H1).

#### Leadership taking charge behavior and employees’ perceptions of organizational change significance

Perception of organizational change significance refers to employees’ awareness and understanding of the importance and necessity of organizational change, as well as their comprehension of change-related information (Woodman, 1995). Employees with a high level of change cognition—defined as a thorough understanding of the value and necessity of change—are more likely to overcome resistance, (Lu et al., 2024) actively accept and adapt to changes, and contribute to their successful implementation. This proactive engagement ultimately enhances work performance and boosts organizational competitiveness (Zhou et al., 2020).

Social learning theory emphasizes that environmental factors and learning processes play a crucial role in shaping individual behavioral cognition, with imitation being a key component during social interactions. Within an organization, leaders act as significant environmental factors, conveying specific information through their behaviors and traits. When employees observe their leaders, their understanding often extends beyond surface-level observation, leading to deeper comprehension and imitation of the conveyed behavioral information. A leader’s taking charge behavior communicates critical information about change, including goals, strategies, anticipated outcomes, and potential challenges. This enables employees to develop a comprehensive understanding of the change, fostering a deeper awareness of its necessity for both personal work and organizational development (Du and Cui, 2019).

Furthermore, constructive change behaviors, such as innovative process development and problem correction, often involve a degree of risk. Leaders who are willing to embrace change typically exhibit a corresponding tolerance for risk and maintain a supportive and encouraging attitude toward organizational change initiatives. In this context, leaders can alleviate employees’ concerns about potential opposition from superiors or threats to their interests during the change process by role modeling and using charismatic appeal. This approach enhances employees’ sense of identity with the change and their understanding of its significance, fostering a deeper appreciation of organizational change.

Therefore, we hypothesize that leadership taking charge behavior has a positive effect on employees’ perceptions of organizational change significance (H2).

### Method

#### Participants and procedure

The questionnaire survey was conducted using paper questionnaires distributed offline. A total of 379 questionnaires were distributed across several institutions, including the Industrial and Commercial Bank of China Zhenjiang Branch, Bank of China Zhenjiang Branch, Panhandle Science and Technology Company, and Rudong City Investment Company. After excluding invalid responses, 316 valid questionnaires were retained, resulting in an effective response rate of 83.4%.

The descriptive statistics for the sample are as follows: The gender distribution was 50.2% male and 49.8% female. Age distribution included 6.0% of respondents aged 25 years or younger, 34.5% aged 26–30 years, 32.3% aged 31–35 years, 17.1% aged 36–40 years, and 10.1% aged 40 years or older. Regarding educational level, 17.8% had specialized education or lower, 59.0% held a bachelor’s degree, 22.9% had a master’s degree, and 0.3% possessed a doctorate or higher. In terms of work experience, 13.9% had less than 1 year, 32.8% had 1–3 years, 28.2% had 4–6 years, 15.0% had 7–10 years, and 10.1% had more than 10 years.

#### Measures

In this study, measurement scales were designed using a 5-point Likert scale, where responses ranged from 1 (“Strongly Disagree”) to 5 (“Strongly Agree”). The specifics of the scales are as follows:

*Leadership Taking Charge Behavior.* This variable was assessed using a scale developed specifically for this study, comprising three dimensions—initiative in change, responsibility, and risk-taking—with a total of nine items. An example item is, “My leader is willing to make more efforts to implement change.” The Cronbach’s *α* for this scale was 0.928.
*Felt Obligation for Constructive Change.* This variable was measured using a unidimensional scale from (Liang et al., 2012), which includes five items such as, “I will do whatever I can to contribute to the organization, such as proposing ideas and solutions to achieve the organization’s goals.” The Cronbach’s α for this scale was 0.851.
*Perception of Organizational Change Significance.* This variable was evaluated using the dimensional scale from (Wu, 2010) Cognition of Change Measurement Inventory, which consists of five items. An example item is, “I believe that the company must make this change, or it may face an operational crisis.” The Cronbach’s α for this scale was 0.902. Additionally, demographic variables were included and controlled for in the regression analysis, as they may influence cognitive patterns and behavioral performance.

### Results

#### Reliability test of the measurement model

Through the validation factor analysis (Table 7), it was found that the fitting indexes of the three-factor model met the requirements of the optimal indexes (χ^2^/df = 2.42, RMSEA = 0.067, CFI = 0.965, IFI = 0.965, NFI = 0.942). Moreover, the three-factor model fitted better than the one-factor and two-factor models.

**Table 7:** Confirmatory factor analysis (*N* = 316).

Model	χ^2^/df	RMSEA	CFI	IFI	NFI
Three-factor model (A, B, C)	2.42	0.067	0.965	0.965	0.942
Two-factor model (A + B, C)	9.797	0.167	0.774	0.776	0.756
Two-factor model (A + C, B)	11.427	0.182	0.732	0.734	0.716
Two-factor model (B + C, A)	9.238	0.162	0.789	0.79	0.77
Single-factor model (A + B + C)	17.219	0.227	0.577	0.579	0.565

A represents “Leadership Taking Charge Behavior,” B represents “Felt Obligation for Constructive Change,” C represents “Perception of Organizational Change Significance.”

Harman’s one-way test revealed that the unrotated first principal component accounted for 39.309% of the total variance, which is notably lower than the empirical threshold of 50%. Additionally, reliability testing for each variable demonstrated that the Cronbach’s alpha coefficients for the Leadership Taking Charge Behavior, Felt Obligation for Constructive Change, and Perception of Organizational Change Significance scales all exceeded 0.7, indicating strong internal consistency within the scales. Validity analysis results (Table 8) showed that the AVE values for all scales were greater than 0.5, and the CR values exceeded 0.8, suggesting that the scales exhibit satisfactory convergent validity. Furthermore, the square root of the AVE for each variable was significantly higher than its correlation with other variables, confirming good discriminant validity. Descriptive statistical analysis also revealed significant correlations between Leadership Taking Charge Behavior and both Felt Obligation for Constructive Change and Perception of Organizational Change Significance, providing preliminary support for the proposed hypotheses.

**Table 8:** Correlation analysis, reliability, and validity testing among variables (*N* = 316).

Category	Leadership taking charge behavior	Felt obligation for constructive change	Perception of organizational change significance
Leadership taking charge behavior	0.856		
Felt obligation for constructive change	0.422^**^	0.811	
Perception of organizational change significance	0.219^**^	0.583^**^	0.748
Average variance extracted (AVE)	0.732	0.559	0.657
Composite reliability (CR)	0.890	0.861	0.905
Cronbach’s α	0.928	0.851	0.902

Diagonal values represent the arithmetic square roots of the average variance extracted (AVE) for each factor. **Indicates *p* < 0.01.

#### Regression analysis

After controlling for gender, age, education, and years of experience, the regression results presented in Table 9 reveal that leadership taking charge behavior is significantly and positively associated with felt obligation for constructive change (Model 2: *β* = 0.336, *p* < 0.001) and with perception of organizational change significance (Model 3: *β* = 0.218, *p* < 0.001). These results support Hypotheses H1 and H2, indicating that the Leadership Change Scale developed in this study demonstrates strong predictive validity.

**Table 9:** Regression analysis results (*N* = 316).

Variable	Felt obligation for constructive change		Perception of organizational change significance	
	Model 1	Model 2	Model 1	Model 2
(Constant)	3.143	1.907	3.546	2.744
Gender	0.154	0.094	−0.022	−0.061
Age	0.166^***^	0.127^**^	0.167^**^	0.141^**^
Education level	−0.016	0.053	−0.102	−0.057
Years of work experience	−0.002	0.039	0.033	0.060
Leadership taking charge behavior	/	0.336^***^	/	0.218^***^
*R* ^2^	0.058	0.202	0.063	0.106
∆*R*^2^	/	0.143	/	0.043
F	4.141^**^	13.489^***^	4.507^**^	6.327^***^

**Indicates *p* < 0.01. ^***^Indicates *p* < 0.001.

## Conclusion and discussion

To explore the conceptual structure of leadership taking charge behavior within the Chinese context, this study employed a mixed-method approach that combined theoretical analysis with empirical examination of the framework underlying leadership taking charge behaviors. Subsequently, a measurement scale was developed. The findings suggest that, within the Chinese cultural context, leadership taking charge is a multidimensional construct encompassing initiative-driven change, responsibility-taking, and risk tolerance, all of which have significant implications. Further analysis confirmed that the scale measuring leadership taking charge demonstrates robust reliability and validity, establishing it as an effective tool.

Existing research has not thoroughly explored the conceptual structure of leadership taking charge behavior within the Chinese context. This study provides both theoretical and empirical contributions to this gap, clarifying the distinctions and connections between leadership taking charge behavior in China and the Western concept of “taking charge.” It also enriches the current literature on taking charge behavior and offers novel theoretical insights for leadership research and practice in China. This study reveals that leadership taking charge behavior in China not only incorporates traits such as “spontaneity,” “change orientation,” and “constructiveness” found in the Western concept of “taking charge,” but also emphasizes additional dimensions. Specifically, it highlights leaders’ sense of responsibility and mission concerning change, as well as their courage and accountability in embracing and taking responsibility for change. This behavior reflects management practices in navigating significant societal changes and offers a unique perspective on leadership behavior within the Chinese cultural context. It aligns with Confucian values, which emphasize “bearing responsibility” and “accountability.”

Confucian culture, rooted in collectivist values, promotes the principle of “righteousness before profit” and stresses that moral responsibility takes precedence over personal gain (Zhang et al., 2013). Leaders are expected to exemplify gentlemanly qualities, adhering to moral norms and obligations that guide their actions. This perspective highlights the importance of leading by example and setting a positive precedent. Thus, Chinese leaders bear heightened responsibility in initiating and sustaining change, particularly in the face of risks and challenges. Additionally, China’s society is characterized by a “relationship-based” approach and a “differential” organizational structure (Fei, 1992), which means that implementing change often carries significant risks. These risks extend beyond policy and institutional levels, affecting deeply rooted relational networks shaped by cultural traditions. As such, resilience in the face of these risks is essential. Organizational leaders must be willing to confront risks and challenge established models to drive effective change, ensuring organizational adaptability and long-term success.

Historically, scales for measuring leadership taking charge behavior were often adapted from the Employee Taking Charge Behavior Scale developed by Morrison and Phelps (1999) within the Western workplace context. However, the differing roles and objectives of organizational leaders and employees during the change process imply that their responsibilities and risk-taking roles vary significantly. In China’s high-power-distance context, ordinary employees typically have limited decision-making authority. They follow the guidance and decisions of leadership during change and bear relatively less responsibility. In contrast, Chinese leaders’ responsibilities during change are multifaceted, encompassing professional, ethical, social, legal, and political dimensions. Western leaders, by comparison, typically have more limited responsibilities, primarily related to business activities, and do not face extensive considerations regarding personal reputation, social stability, or political factors. As such, the reliability and validity of applying Western scales designed to measure employee taking charge behavior to assess leaders’ taking charge behavior within the Chinese cultural context is questionable. To address this gap, this study developed a scale specifically designed to measure leadership change and taking charge behavior within the Chinese context. This new scale not only expands research on change and taking charge behaviors across various subjects, but it also addresses the lack of appropriate measurement tools for leadership taking charge behavior in China, establishing a foundation for future empirical research.

### Practical implications

From the perspective of enterprises, assessing and cultivating leaders’ behaviors should involve multiple aspects, with a focus on fostering their initiative and sense of responsibility. It is essential to encourage leaders to act decisively and take responsibility by enhancing their subjective awareness of “taking responsibility” and their courage to “not fear risks.” Specifically, enterprises should concentrate on developing leaders’ sense of responsibility, their resilience to risk, and their ability to drive change, thereby transforming their awareness of change into actionable behaviors. Additionally, enterprises should provide the necessary resources and conditions to support leaders in assessing and implementing change measures and create ample opportunities and platforms for leaders to engage in change initiatives.

From the leader’s perspective, it is crucial to act as a role model for subordinates, demonstrating courage in embracing change and actively undertaking responsibilities. Leaders should exhibit initiative and a responsible demeanor, using their example to promote the effective implementation of change measures. This approach will inspire the team to progress collectively, foster a culture of change within the enterprise, and sustain momentum for continued development. Ultimately, this will enhance the leader’s ability to guide subordinates and drive the organization toward positive growth.

### Limitations and future research directions

Despite the adherence to rigorous procedures, this study has certain inevitable limitations. Firstly, the survey sampling was not fully randomized, and the survey locations were predominantly concentrated in Jiangsu, Anhui, and Shanghai. This geographic concentration may limit the applicability of the conclusions. Future research should aim to conduct a nationwide survey to collect data from diverse geographic regions, thereby ensuring a more balanced distribution and enhancing the generalizability of the findings. Secondly, the Leadership Change Behavior Scale developed in this study is a self-assessment tool, with subordinates evaluating their leaders. While this is common practice in leadership research, it may introduce measurement errors due to subordinates’ emotions, attitudes, and social expectations, which can affect result accuracy. Future studies should consider using a combination of self-assessment and other assessment scales to mitigate the impact of subjectivity and improve the accuracy and reliability of evaluations.

## Data Availability

The raw data supporting the conclusions of this article will be made available by the authors, without undue reservation.
